# Supplementary figures and images for: Simpler predictive models provide higher accuracy for ovarian cancer detection
Source: PeerJ. 2025 Dec 18;13:e20525. doi: 10.7717/peerj.20525 (PMC12716435; doi:10.7717/peerj.20525)

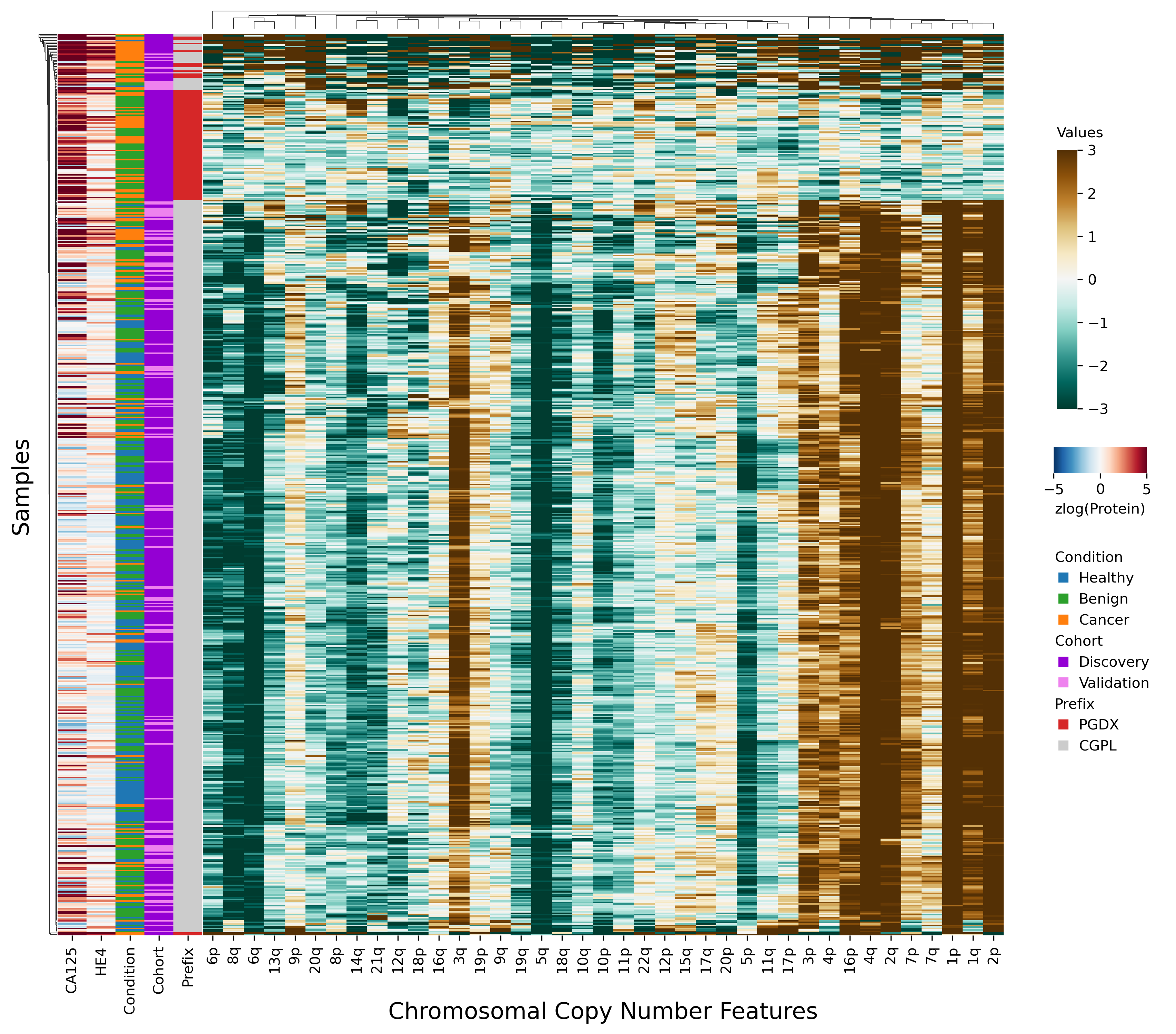

Supplement: Supplemental Information 1 — The samples from the discovery cohort are shown here, with each row representing features from a single sample. [file peerj-13-20525-s001.png]

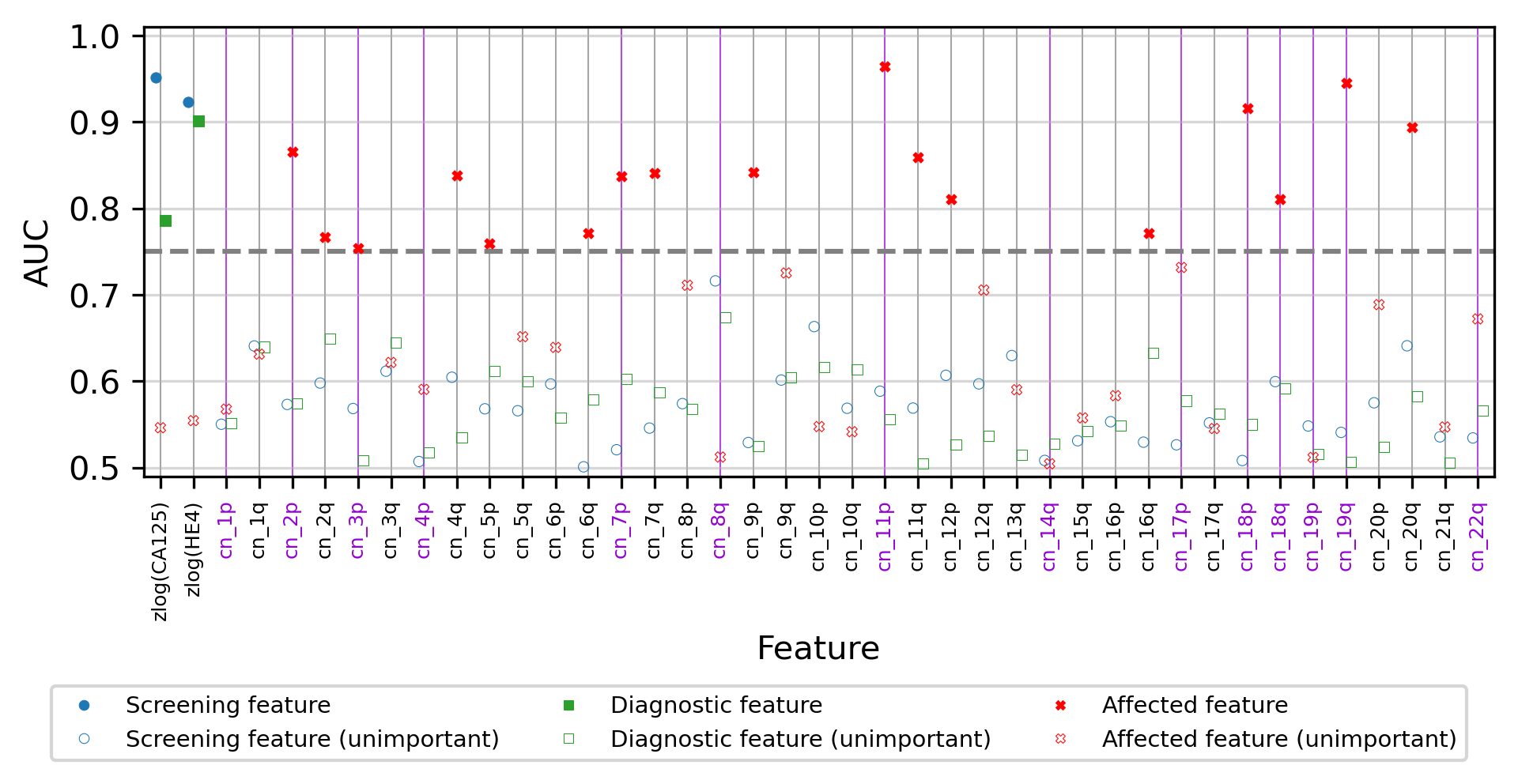

Supplement: Supplemental Information 2 — For each displayed feature, we analyzed the discovery cohort and computed three receiver operating characteristic (ROC) curves and measured the area under the curve (AUC). Two curves were calculated on CGPL-prefixed samples, measuring a feature’s association with cancer in a screening and a diagnostic context. The third curve was calculated on cancer samples only, and measured a feature’s association with the two different prefixes (CGPL and PGDX) to determine how affected a feature was by batch confounding. Features with a “cn_” prefix are copy number features, and those with a “zlog” prefix are the zlog-transformed protein concentrations; copy number features listed in the DELFI-Pro results as contributing to the DELFI-Pro screening model performance are highlighted in violet. We use an AUC threshold of 0.75 to separate features that are good predictors from those features that are less important; features described as “unimportant” in the figure are those with AUC values less than 0.75. [file peerj-13-20525-s002.png]

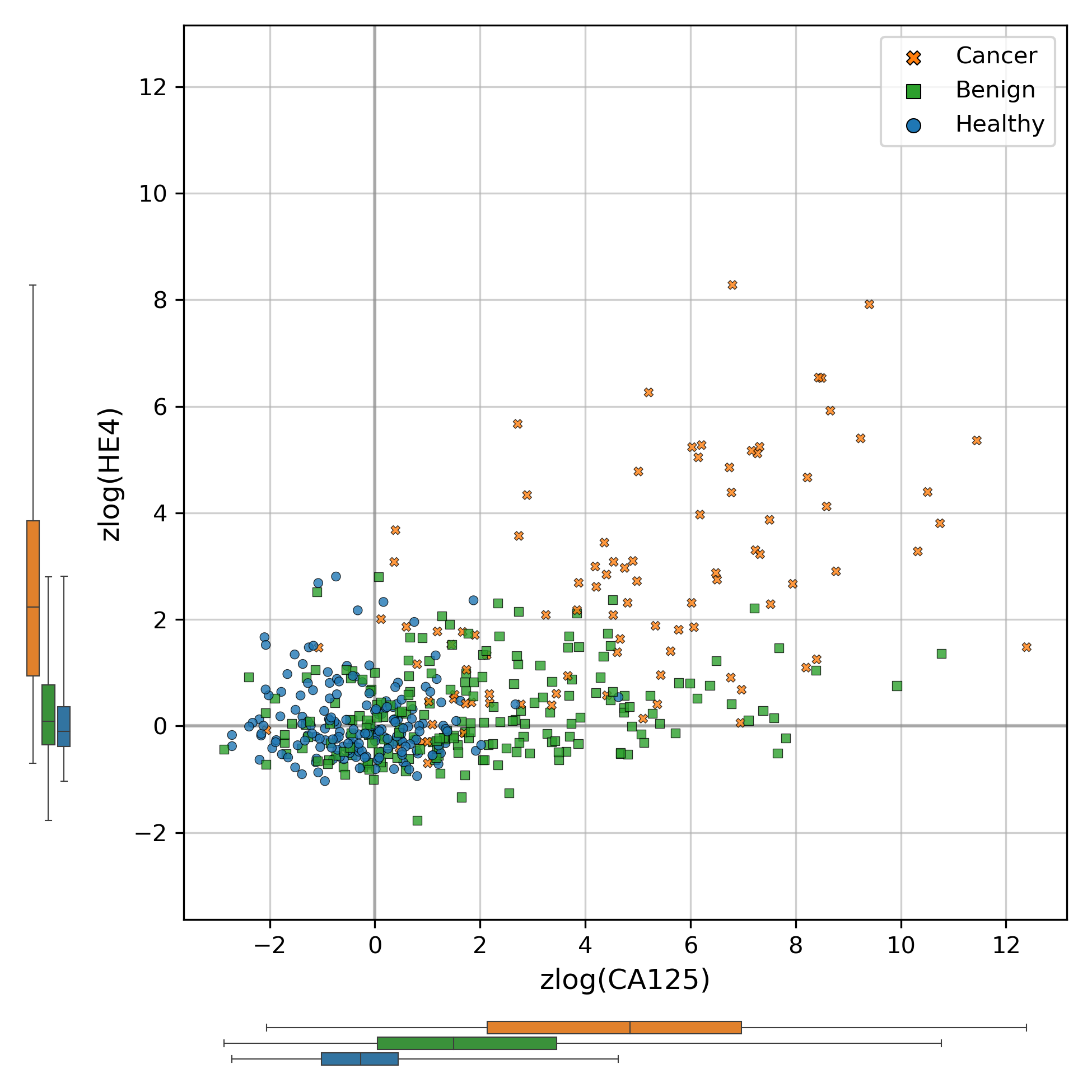

Supplement: Supplemental Information 3 — Marginal distributions of the two protein levels within the three conditions of interest are shown to the left and under the corresponding axes, with boxplots indicating the interquartile range and whiskers representing the range from the minimum to maximum values. [file peerj-13-20525-s003.png]

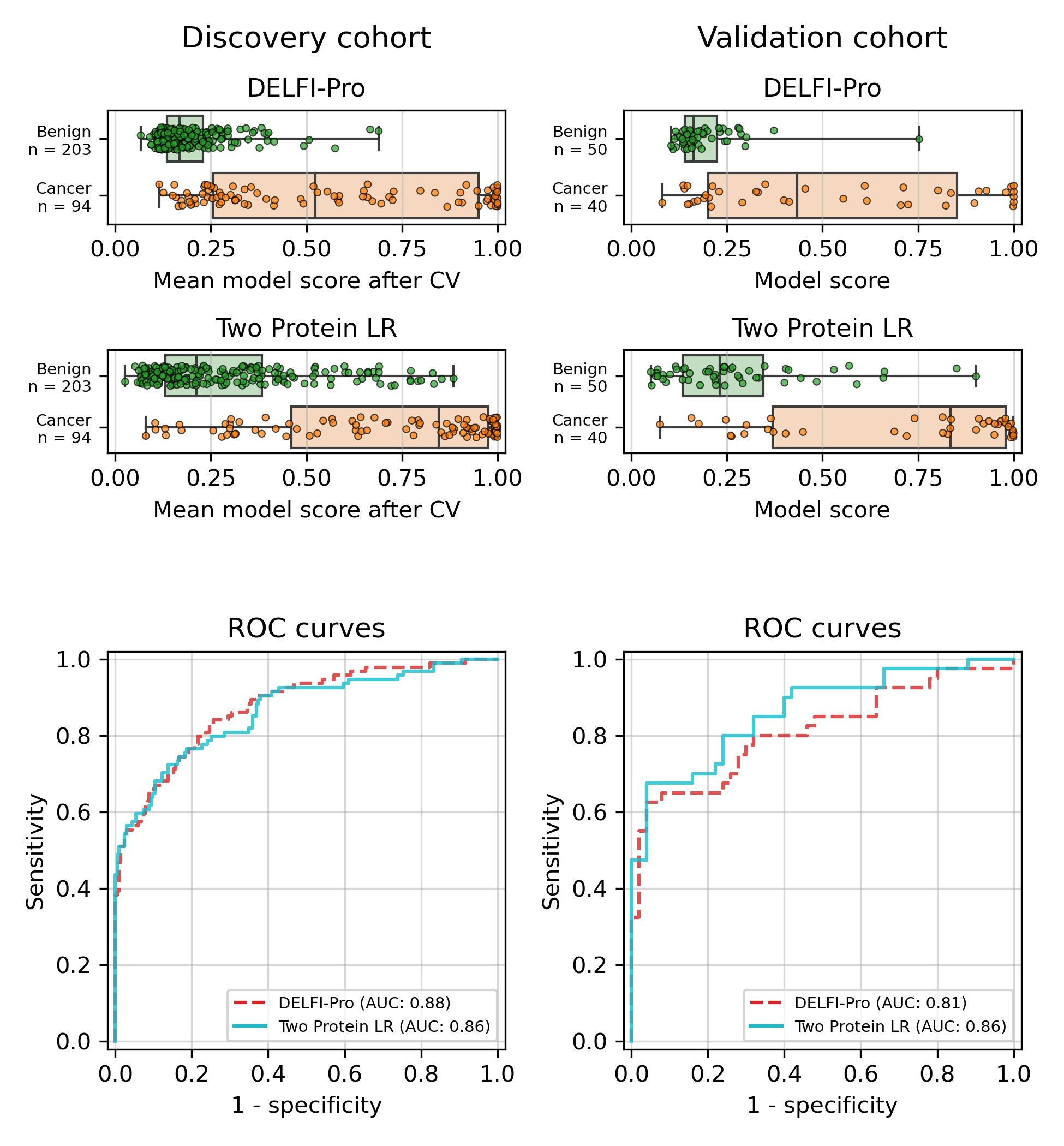

Supplement: Supplemental Information 4 — Cross validation (“CV”) results for the discovery cohort, and validation results on the validation cohort are shown for both the DELFI-Pro diagnostic model and a two-protein logistic regression (“Two Protein LR”) diagnostic model. Both models seek to distinguish between samples from donors with benign adnexal masses and samples from patients with ovarian cancer. Receiver operating characteristic (ROC) curves demonstrate the performance of the two models at various score thresholds, with the area under the curve (AUC) growing proportionally to model accuracy. [file peerj-13-20525-s004.png]

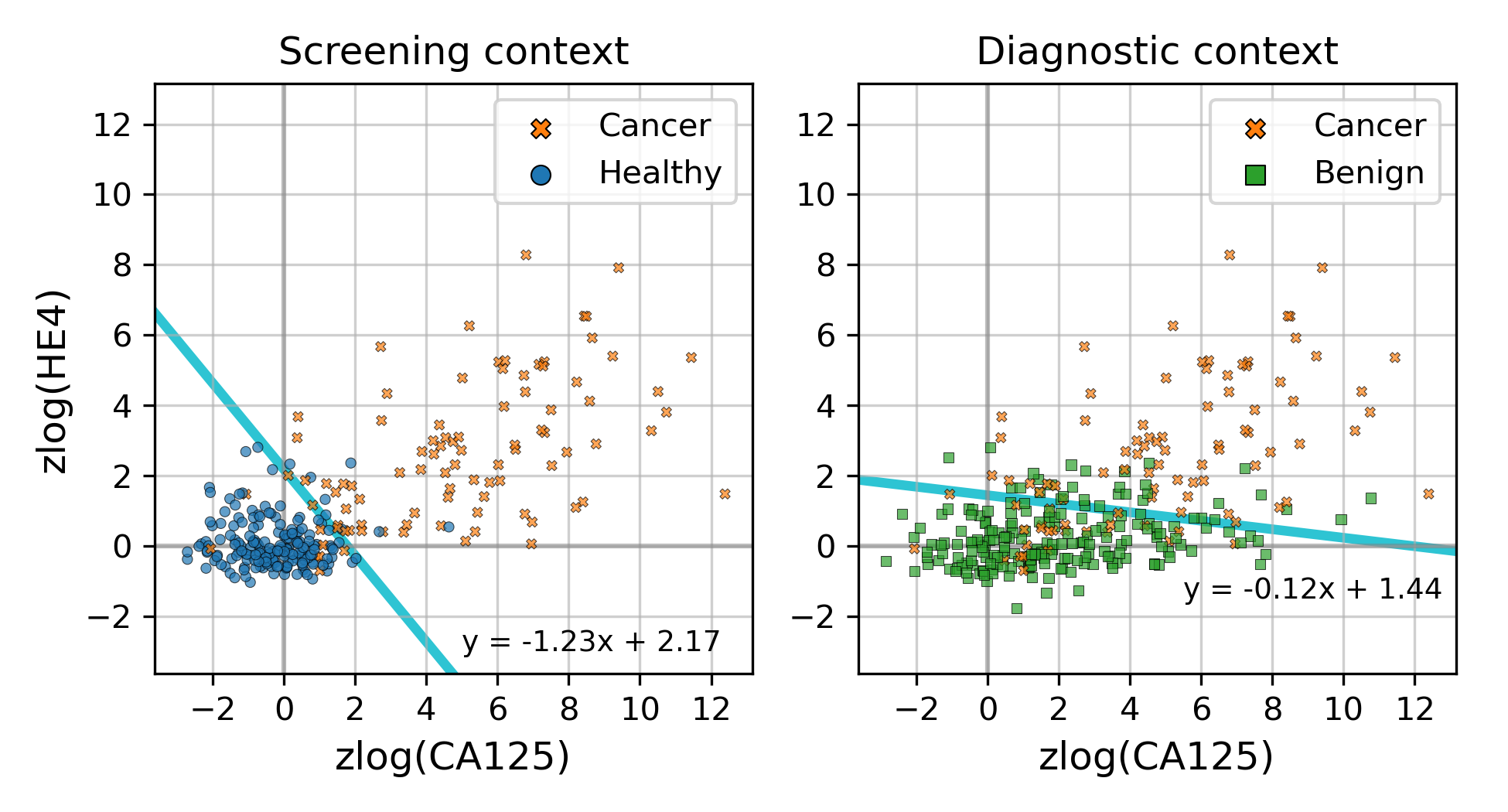

Supplement: Supplemental Information 5 — On the discovery cohort, two protein-only models were fit to the data. In the screening context, the model distinguishes between samples from healthy donors and samples from patients with ovarian cancer. In the diagnostic context, the model distinguishes between samples from donors with benign adnexal masses and samples from patients with ovarian cancer. The decision boundaries for the two models are drawn in cyan, and equations of the decision boundary lines are written near the lines. [file peerj-13-20525-s005.png]

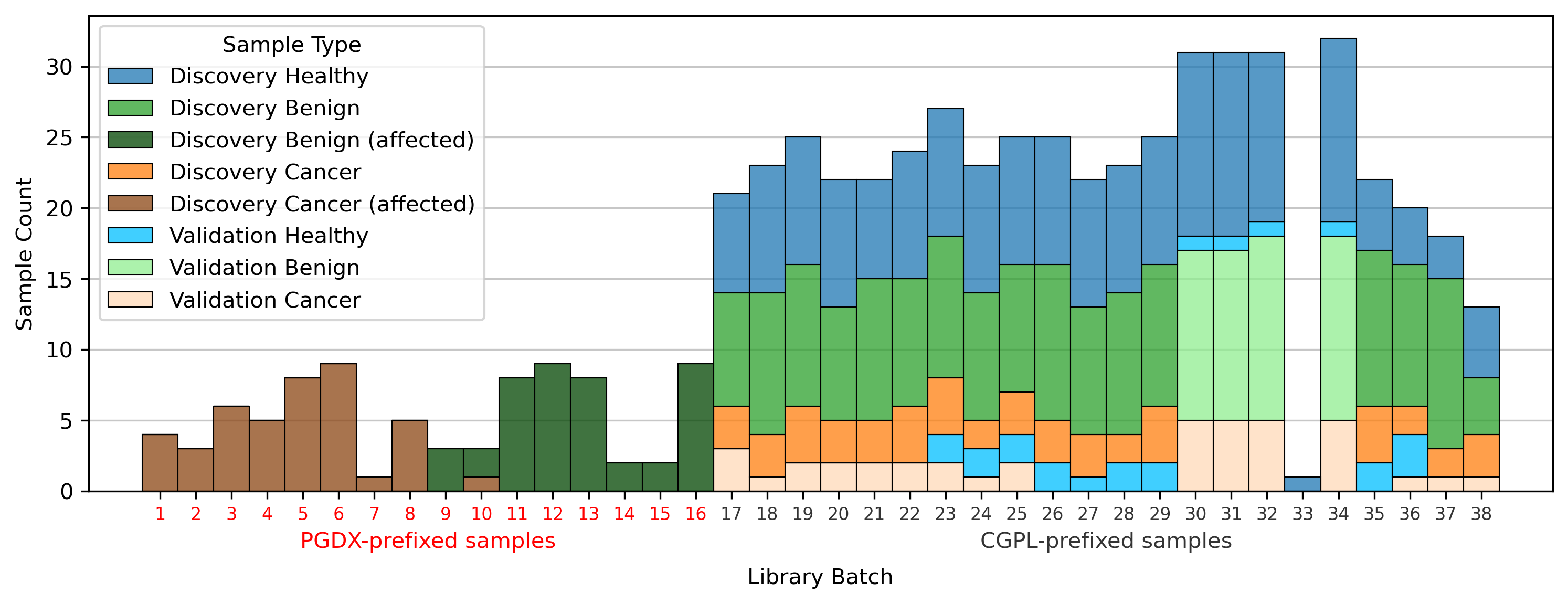

Supplement: Supplemental Information 6 — All samples used as part of the DELFI-Pro publication’s dataset are divided into three conditions (healthy, benign, and cancer), and into two cohorts (discovery and validation). Samples with the “PGDX” sample prefix-associated effect (labelled “affected”) are colored separately. The 16 batches that only have affected samples are colored red, with the other batches that only have CGPL-prefixed samples colored gray. [file peerj-13-20525-s006.png]
